# A Comparatively Safe If Not New Operation for Varicocele

**Published:** 1883-04

**Authors:** R. G. Bogue

**Affiliations:** Chicago


### ARTICLE IV.

### A COMPARATIVELY SAFE IF NOT NEW OPERATION FOR VARICOCELE.—By R. G. BOGUE, M.D., Chicago.

Fred. Horner, aged 11 years, a loose fibered, thin skinned lad, but not delicate, has had enlargement of the left side of the scrotum for several years, which has gradually increased in size. The scrotal veins of left side are very large and tortuous and the skin of scrotum very thin ; has for a long time worn a suspensory bag, which has afforded but little relief, and has not prevented the steady enlargement.

From considerations which will be given further on, I was led to decide upon the plan of operation adopted in this case, which to me is new. It may be an old and discarded operation, but from having given some thought to the subject of "radical cure operation" for varicocele, I believed that this operation contain elements of safety which none of the list of recognized operations for varicocele do not.

March 19, 1880, with the assistance of Dr. John Bartlett, and using the carbolated spray, I made an incision over the lower part of the cord and upper part of the scrotum, about one and a half inches in length, exposing the veins, and by use of an aneurism needle ligated the veins at four points with fine plaited silk ligature, which had been thoroughly carbolized, tied tightly and cut the ends close to the knot. I supposed that I tied two veins, each at two points, left a small drainage tube in the lower end of the wound and closed it with horse hair sutures, covered the parts well with carbolated gauze and supported the whole scrotum with a pad between the thighs so that there should be no dragging upon the cord. On the second day the drainage tube was removed, and the sutures were removed on the second or third day following. The wound healed very kindly, without a drop of suppuration. The dressings were changed each day and the parts bathed or cleansed with carbolated water. There was left an indurated and tender mass, where the ligatures were, about the size of the last joint of the little finger. After two weeks the boy was allowed to be about; the scrotum supported by a suspensory bag. At first it seemed that little or nothing had been gained. The veins were as large and numerous in the scrotum below the point of ligature as before. But the tenderness and induration began to disappear, and finally the parts became entirely supple and the scrotum about one-half its former size. The ligatures could be felt as little hard bodies, but causing no annoyance. One or two veins could be followed along the cord.

On July 31, 1880, with the assistance of Doctors Bartlett, Parkes and Hooper, I cut down upon the veins at the point of previous operation, and applied three fine silk ligatures, two around one vein, about one-third to half an inch apart, and one about the other. The wound was closed and dressed as before, and it healed as kindly. The induration at point of ligation disappeared faster, leaving no veins to be felt in the scrotum, showing that all of the veins had been obliterated, and now, about four months after the second operation, the left side of the scrotum is no larger than the other; there are no large veins, and the testicle is firmer and larger than before the operation. The several ligatures can be felt as little firm bodies, but no tenderness about them. They have in this case proved to be entirely inoffensive to the tissues, having served the purpose for which they were used and then the tissues immediately about them not being irritated by their presence.

Radical cure for varicocele consists in destroying the main scrotal veins as blood channels. This has been accomplished by ligation in open wounds, by subcutaneous ligation with silk and metal, by compression with needles, pins and wires variously applied. By these methods the veins and all other tissues included within the appliance are to be cut through by ulceration. Another method is compression of the vessels by tying over the ends of needles thrust through beneath them. The needles and ligatures are left in place until sufficient inflammation has been provoked to obliterate the veins. Whether this is uniformly accomplished, or whether attended by any unpleasant or unfortunate results, I am unable to say. Another operation which has been resorted to is that of cutting away a goodly portion of the skin of the scrotum, that by such retrenchment the scrotal contents should be held up, a kind of substitute for a suspensory bag.

Most, if not all, of these methods have been and are still considered dangerous operations. Dangerous to the patient from gangrene, suppurative phlebitis, septicæmia and pyemia.

Deaths from these have happened often enough to lead most of the authors to be very chary in recommending radical cure operation, if it can be avoided. One says "Don't operate unless the patient urges it upon the surgeon." Operation for varicocele has been known to be dangerous to the surgeon, for Delpeck, a famous French surgeon, was assassinated by a man on whom he had operated for varicocele of both sides, the man believing that the operation was the cause of his loss of sexual desire. The particular operation in this case is not mentioned.

It is proper to inquire into the causes of danger in this operation. Does it depend upon some pecularity of the structures operated upon, or is the suppurative inflammation or gangrene, with its septicæmia and perhaps pyemia due to the kind of operation resorted to, or in other words, is it due to the manner in which the veins and the neighboring tissues have been treated ?

Operation upon large veins in their continuity, has been considered more or less dangerous ; but they give no special anxiety when encountered in an open wound. Under such circumstances they are either left to themselves, or if inclined to bleed, are ligated either one end or both, as the case may suggest, the wound of the vein, or the ligation thereof, seldom being accused as the cause of any untoward results which may follow. As to the parts implicated and the operation under consideration, we are inclined to believe that the fault lies in the kind of operation, instead of any pecularity of the parts. The parts are peculiar, for the contents of the scrotum receive their blood supply by a limited number of long slender arteries, and the blood is returned by only a few long veins, and it is an easy matter to cut off nearly all the supply of blood and also prevent almost wholly the return flow. And really this is what is accomplished by the operation, which has the endorsement of all the authorities.

A needle is thrust through at a point selected in the very upper part of the scrotum, or lower part of the cord in front of the vas deferens and behind the veins, hoping to leave the arteries also behind the needle; another needle is to be passed in front of the veins, and by means of wires attached to the needles to hold them in apposition, they are rolled and in this way the parts included are to be compressed by twisting, or a ligature of silk or wire is to be passed through in the place of the needles and the tissues included strangulated by tying or twisting, or by means of a needle behind and a ligature tied over the ends, all of the tissues in front and a portion of skin are included in the strangulation. Now what has been included within the strangulating appliance besides the enlarged veins which are alone the offending bodies? Nearly everything making up the cord at point of operation except the vas deferens; nearly all of the vessels for supply, nearly all the channels of return, the connective tissue, fascia, cremaster and nerve filaments. By strangulating this mass, the parts beyond are certainly put into about as favorable condition for sloughing as could well be done, excepting the old rude way of treating bucks, which was by tying the whole appendage at the top of the scrotum. Gangrene is as likely to follow the sudden arrest of flow of venous blood from a part as to cut off the supply of arterial blood, particularly so in a distant and dependent part. We are disposed to believe that there is safety in cutting off the channels of blood return, not all at once, but by two operations. If there are three or four enlarged veins, cut down upon them at the upper part of the scrotum; expose and ligate separately each vein, leaving at least one. I think it full better to ligate them at two points, half an inch or so apart; by so doing, a section of the vein will be obliterated. In this way, all of the venous flow is not arrested, only the major portion. Some inflammation will follow, but the parts beyond are not endangered. While recovery from this operation is going on, there will be established collateral circulation sufficient to allow a second operation, ligation of the remaining enlarged vein or veins, with equal safety. The second operation should not be made until all induration from the first has disappeared. And, if the operations are made with the antiseptic precautions, and thus treated, I believe that all of the special dangers attending radical cure operation for varicocele are done away with. Certainly, if there is danger, it is the minimum amount, and with less suffering than by any other operation. Catgut ligatures would, no doubt, answer just as well as silk.

I operated upon a case, several years ago, where the scrotum was much elongated, the skin thin, the veins large, and the patient an adult, by what is called the Henry Lee plan, which consists in subcutaneous ligation of the veins at two points, about one inch apart, and subcutaneous division of them between the ligatures. It was followed by gangrene and sloughing of the testicle and scrotum of that side, and an attack of septicæmia, which, for a few days, endangered the life of the patient. From the loss of tissue and cicatrization there was thorough retrenchment, and the man had no more pendulous scrotum to annoy him; but it was secured at no little risk.

This case led me to examine the subject, to see whether there might not be found some operation which would be less likely to endanger the parts, and, we believe, that the operation herein advised, to be more reasonable and less dangerous than any other, for only the veins are ligated, leaving all the other structures uninjured, the flow of blood to the parts not interfered with, and the venous flow not wholly arrested at one time; all of which must tend to secure the minimum amount of injury and risk.

